# Determination of Zalcitabine in Medicaments by Differential Pulse Voltammetry

**DOI:** 10.1155/2013/495814

**Published:** 2013-04-04

**Authors:** Katia Christina Leandro, Josino Costa Moreira, Pércio Augusto Mardini Farias

**Affiliations:** ^1^Fundação Oswaldo Cruz, Rio de Janeiro, RJ, Brazil; ^2^Department of Chemistry, Pontifícia Universidade Católica, 225 Rua Marquês de São Vicente, 22453-900 Rio de Janeiro, RJ, Brazil

## Abstract

The zalcitabine (ddC) has been extensively used in the treatment of HIV patients due to its antiretroviral activity. The quality control of this active principle in medications is of outstanding importance to public health. The principal objective of the current study was the development of an alternative analytical methodology for the zalcitabine determination using a voltammetric process. The zalcitabine gives a reduction peak (at −1.22 V versus Ag/AgCl) at the hanging mercury drop electrode (HMDE). The differential pulse voltammetric response is evaluated with respect to the scan rate (20 mV/s), pulse amplitude (50 mV), support electrolyte (Clark-Lubs buffer), pH (2.0), and other variables. The response is linear over the 10.0 to 28.0 mg/L (47 to 133 *μ*M) concentration range, and the detection limit is 2.08 mg/L. The validation of this method was realized using a governmental Brazilian document (Inmetro, 2007) and the results are reported for medication drugs.

## 1. Introduction

**Figure 1:**
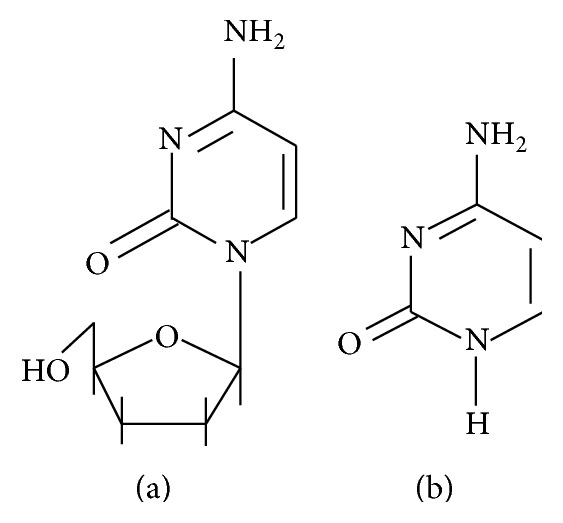
Structures of (a) zalcitabine and (b) cytosine.

The Food and Drug Administration has approved the AIDS drug zalcitabine, commonly known as 2′-3′-dideoxycytidine (ddC). It is the third drug licensed specifically for use in treating the human immunodeficiency virus, the cause of AIDS. Zalcitabine was approved for use in combination with the first approved AIDS drug, zidovudine (or AZT), as a treatment option for adult patients with advanced HIV infection who show signs of clinical or immunological deterioration. Early clinical trial data have shown that increases in CD4 cells (immune cells) were somewhat greater and more sustained in patients treated with the combination of zalcitabine and zidovudine than in those who received zidovudine alone as initial therapy. An increase in CD4 cells is believed to indicate that the body's disease-fighting capability has been at least transiently enhanced. Zalcitabine is a derivative of the naturally existing deoxycytidine, made by replacing the hydroxyl group in position 3′ with a hydrogen group. It is phosphorylated in T cells and other HIV target cells into its active triphosphate form, ddCTP. This active metabolite works as a substrate for HIV reverse transcriptase, and also by incorporation into the viral DNA, hence terminating the chain elongation due to the missing hydroxyl group. Since zalcitabine is a nucleoside, reverse transcriptase inhibitor (NRTI), it possesses activity only against retroviruses [1, 2]. Zalcitabine has a very high oral absorption rate of over 80%. It is predominantly eliminated by the renal route, with a half-life of 2 hours [3, 4]. Zalcitabine (Figure 1(a)) is an analog of cytosine (Figure 1(b)). The base cytosine is a pyrimidine present on DNA.

Several chromatographic methods for the determination of zalcitabine in human plasma or serum have been published [5–8]. These assays utilize a variety of techniques including the flow-injection spectrophotometric technique [9]. Recently, electroanalytical methods for similar zalcitabine antiretroviral drugs for lamivudine determination in human serum [10] and for zidovudine in pharmaceutical dosage forms [11] were also developed. The present work reports preliminary investigations on the electrochemical behavior of zalcitabine in Clark-Lubs buffer electrolyte (pH 2.0) at hanging mercury drop electrode (HMDE) and proposes an alternative approach using differential pulse voltammetry to the determination of trace amounts of zalcitabine in medicaments.

## 2. Experimental

### 2.1. Apparatus

Cyclic and differential pulse voltammograms were obtained with a Metrohm Voltammetric System, Model 757 VA Computrace. The working electrode was a hanging mercury drop electrode (HMDE), an Ag/AgCl as reference electrode, and platinum as an auxiliary electrode. All pH measurements were made with a Micronal B474 pH Meter using combined Ag/AgCl-glass electrode.

### 2.2. Materials and Reagents

Water purified in a Milli-Q system (Millipore) was used for all dilutions and sample preparations. All chemicals were of analytical reagent grade.

Stock solutions of zalcitabine (229 mg · L^−1^) were prepared daily by dissolving 22.9 mg of zalcitabine in water and a diluting to 100 mL with water. Zalcitabine standard was used as received by the “Instituto Nacional de Controle de Qualidade em Saúde” (INCQS) from Brazil (lot L1, 100.2%). Capsules of medicament containing 0.750 mg of zalcitabine were dissolved with water, filtered, and transferred into a 50.0 mL volumetric flask. The solutions were stored in the dark at 4°C. The 0.2 M Clark-Lubs buffer (pH 2.0) was also prepared in water. The pH of 0.04 M Britton-Robinson buffer (pH 2.0–11.0) was adjusted by addition of hydroxide sodium solution.

### 2.3. Procedure

10 mL of the supporting electrolyte solution (0.2 M Clark-Lubs buffer, pH 2.0) was added to the voltammetric cell and degassed with nitrogen for 2 min while being stirred. An initial potential −1.00 V was applied to the electrode and the stirring was then stopped and the voltammogram was recorded by applying a negative-going potential scan (with differential pulse mode). The scan (at 20 mV/s and 50 mV pulse amplitude) was terminated at −1.25 V. After the background differential pulse voltammograms had been obtained, aliquots of the zalcitabine (0.25 mL of the stock solution) were added. Analysis of the medicaments forms followed the same procedure. In this case, 0.50 mL of the drug solution was used and the differential pulse cycle was repeated ten times using a new mercury drop. All voltammograms were recorded at ambient temperature (25°C).

## 3. Results and Discussion

### 3.1. Cyclic Voltammetric Studies

The effect of scan rate on the peak current (*I*_*p*_) and potential (*E*_*p*_) of zalcitabine was studied by cyclic voltammetry. For both cases, an increase in scan rate caused an increase in *I*_*p*_ with a simultaneous negative shift of *E*_*p*_.

**Figure 2:**
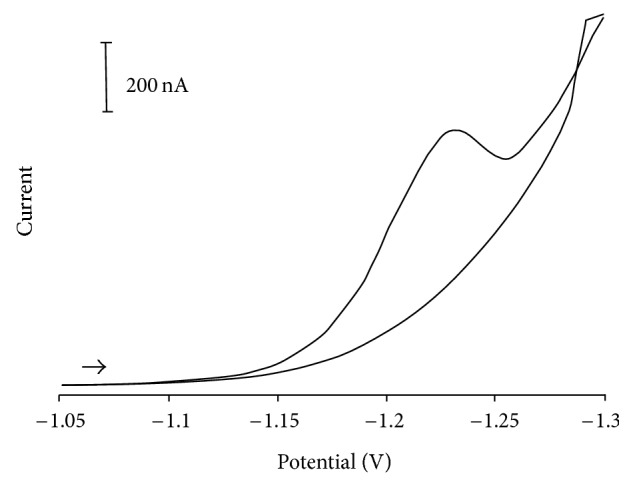
Cyclic voltammogram of the zalcitabine (20 mg · L^−1^) in 0.2 M Clark-Lubs buffer (pH 2.0). Scan rate: 100 mV s^−1^.

Figure 2 shows a typical cyclic voltammogram for 20 mg · L^−1^ zalcitabine in a 0.2 M Clark-Lubs buffer solution (pH 2.0) using 100 mV/s as scan rate. The zalcitabine yields a well-defined reduction peak at −1.22 V during forward cathodic scan. No peaks are observed in the anodic branch indicating irreversibility of the electrodic reaction. Similar behavior was observed at pH below 5.5. In this range of pH no adsorption of zalcitabine in the mercury electrode surface was observed. Hence, the reduction peak at –1.22 V without accumulation was chosen for subsequent differential pulse voltammetric determinations, including the quantification of zalcitabine in antiretroviral drugs.

### 3.2. pH Dependence

The influence of pH on the zalcitabine reduction process was studied by differential pulse voltammetry. The Britton-Robinson buffer solution was used in the range 2.0 to 11.5 pH and the zalcitabine reduction peak again appeared as in cyclic voltammetry only from 2.0 to 5.5 pH.  Table 1 shows that the zalcitabine peak current is maximal from 2.0 to 3.0 pH. These results suggest the importance of an acid medium for production of possible intermediates in the mechanism of zalcitabine electrochemical reaction. The effect of the other different buffer solutions as acetate and Clark-Lubs buffer (in the zalcitabine peak) was also tested. The 0.2 M Clark-Lubs buffer solution (pH 2.0) showed better compromise between sensitivity and resolution and was used further throughout the study. The pH also affects the peak potential (*E*_*p*_, Figure 4). When increasing the pH values from 2.0 to 5.5 a cathodic shift of the zalcitabine potential peak was observed. These results are most likely indicative of the participation of protons in the electrodic process. The slope obtained for this linear relation was of 56.7 mV pH^−1^ indicating the consumption of an identical number of protons and electrons.

**Figure 3:**
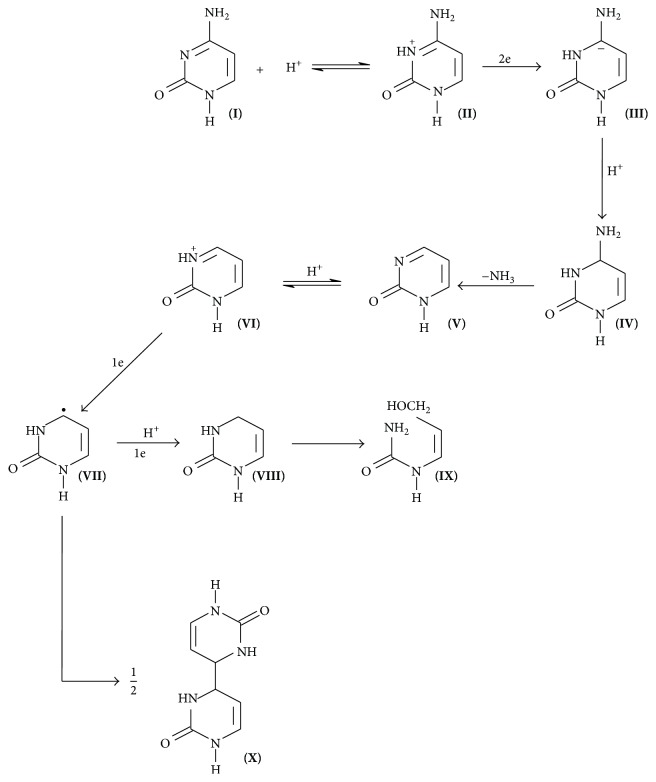
Mechanism of cytosine reduction [12].

A new experiment was realized for a better comprehension of zalcitabine mechanism on the mercury surface. When an aqueous zalcitabine (10 mg · L^−1^) or cytosine solution is electrolyzed, the formation of ammonia could be observed in cases. The presence of ammonia was proved by Nessler's reagent forming a brown coloration or precipitate. These results and the similar structure of zalcitabine with that of cytosine are indicative that the reduction of the zalcitabine mechanism could be practically the same as proposed previously for cytosine (Figure 3). 

### 3.3. Quantitative Application

**Figure 4:**
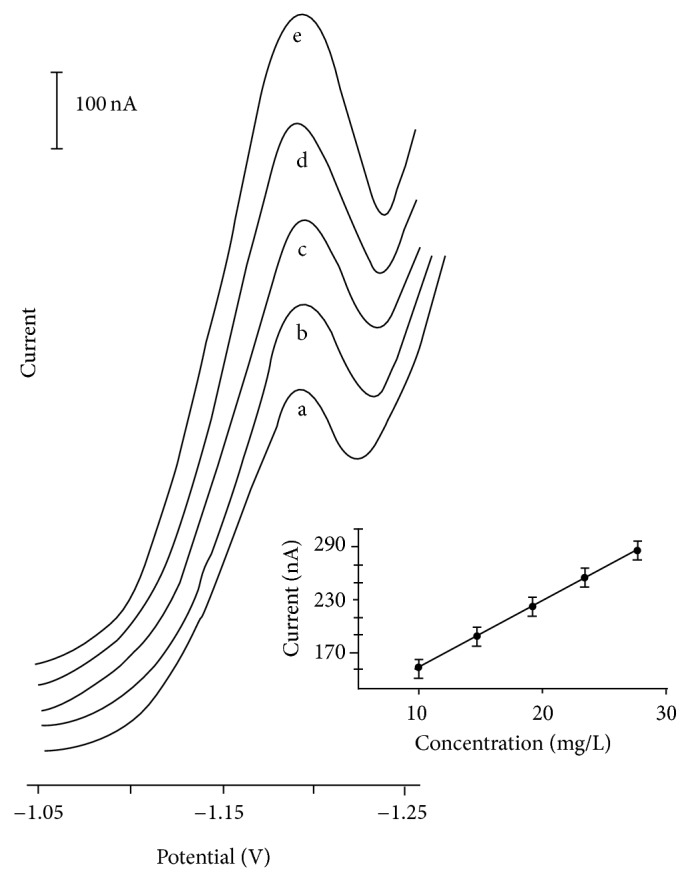
Differential pulse voltammograms obtained for solutions of increasing zalcitabine concentration, 10.0–28.0 mg · L^−1^ (a–e). Other conditions as in Table 1. Resulting calibration plot based on the data for (a)–(e) is in inset.

Figure 4 illustrates the response to successive standard additions of zalcitabine, each addition corresponding to a 4.50 mg · L^−1^ increase in concentration. Well-defined differential pulse voltammetric peaks are observed at the 10.0–28.0 mg · L^−1^ concentration level. The resulting plot of peak current versus concentration (also shown in Figure 4) is linear (slope 7.73 nA/mg · L^−1^; correlation coefficient, 0.999). The differential pulse voltammetric response of zalcitabine is highly reproducible. Five successive measurements for each point of the analytical curve yielded standard deviations of 0.085, 0.174, 0.232, 0.130, and 0.268, respectively. A detection limit of 2.08 mg · L^−1^ and a quantification limit of 4.12 mg · L^−1^ were found.

### 3.4. Validation of the Method

The developed differential pulse voltammetric method was validated according to international guidelines for bioanalytical methods including stability of analyte, determination of specificity and selectivity, calibration curve, detection and determination limits, accuracy, and intraday precision [13]. The results obtained also were analyzed by statistical calculations [14]. 

For verification of zalcitabine stability, aliquots from stock solution of zalcitabine (229 mg · L^−1^) in 2 M HCl at 25°C, prepared two weeks previously, were added in an electrochemistry cell containing the 0.2 M Clark-Lubs buffer solution (pH 2.0) and analyzed by differential pulse voltammetry. The voltammetric results obtained were compared with those from the stock solution of zalcitabine measured immediately after its preparation.

The specificity and selectivity studies describe the extent to which a method uniquely reacts to a selected element. Seven samples were prepared, in parallel, containing a matrix (e.g., cellulose, starch glycolate sodium, magnesium stearate, hydroxypropylmethylcellulose, titanium dioxide and polyethylene glycol) with 210 mg · L^−1^ zalcitabine (group 1) while seven samples containing purified water with 210 mg · L^−1^ zalcitabine (group 2) were analyzed. The results obtained were compared using *F* tests (homogeneity of variances between groups 1 and 2) to verify if the results showed no considerable differences amongst themselves (relative at precision) and test (comparison of averages between groups 1 and 2) to verify possible systematic errors and to verify accuracy of results. Interactions with zalcitabine and matrix components were not detected.

The major sources of interferences would likely be coexisting ions and organic surfactants. The presence of these species could result in either new reduction peaks or the overlap with the zalcitabine peak thus interfering with the measurement. If this is the case, then selectivity studies must be performed in order to investigate the effect of potentially interfering ions and compounds. The presence of zidovudine (AZT) and lamivudine (3TC) compounds were investigated as possible interferent in the zalcitabine (100 mg · L^−1^ in pharmaceutical form) determination. The presence of lamivudine (3TC) enhanced the zalcitabine peak. The zidovudine (AZT) yields a well-defined reduction peak at −0.96 V (far zalcitabine peak) and lamivudine (3TC) at −1.16 V (near zalcitabine peak) versus Ag/AgCl during forward cathodic scan. The effect of Fe^3+^, Cu^2+^, Zn^2+^, Li^+^, Pb^2+^, Cd^2+^, Ni^2+^, Co^2+^, and Cr^3+^ ions in the free zalcitabine solution was also verified. Concentrations of 100 mg · L^−1^, Fe^3+^, Cu^2+^, Zn^2+^, Li^+^, Pb^2+^, Cd^2+^, Ni^2+^, Co^2+^, and Cr^3+^ did not interfere with the determination of 23 mg · L^−1^ zalcitabine. 

Another interest in studying the presence of ions in the development of this methodology for the determination of zalcitabine was to verify the possibility of complexing zalcitabine with these ions in order to further separate its voltammetric peak potential for better resolution in analytical determinations simultaneously with other drugs antiretrovirals, but no signs of formation of these complexes were detected.

Although voltammetry is well known for benefiting from a wide linear concentration range, this parameter was evaluated by checking the linearity from a set of standard zalcitabine solutions at five levels of concentrations. The differential pulse voltammograms from −1.00 to −1.25 V for each standard solution were obtained with the increasing addition of five aliquots (4.50 mg · L^−1^) of zalcitabine concentration (other conditions: Clark-Lubs buffer, pH 2.0) as supporting electrolyte, a scan rate of 20 mV s^−1^ and a pulse amplitude of 50 mV. The linearity results were analyzed by linear regression and the homoscedasticity by the Cochran test. Using the same conditions the detection and determination limits of zalcitabine in pharmaceutical form were experimentally determined as the lower concentration detected or measured (results in Table 2). 

Repeatability was calculated using the current peak of seven successive measurements from independent sets of standard zalcitabine solution. These results were obtained in the same laboratory by the same operator using the same equipment in a short interval of time. The lower relative standard deviations obtained could also be attributed to the use of a new drop of a reproducible area of a mercury electrode in each run (results in Table 2). 

Intra-day precision of the method was evaluated by assaying seven replicate samples of zalcitabine (150 mg · L^−1^) by the same operator using the same equipment in a long interval of time (two days). The results were available using the Cochran test (to check the variance homogeneity in each day), *F* (to check the compatibility among the variances from each day), and “*t*” (to check if the average from each day belong to the same population) tests (results in Table 2). 

The accuracy was determined by the addition of zalcitabine standard solution at three different concentrations. The used concentrations were near the quantification limit and at medium and maximum points of the calibration curve. The mean of the recovery was calculated using the results obtained from the multiple determinations. After method development and validation were realized, the following aspects of robustness were investigated: the effect of pH (1.5, 2.0, and 2.5) and purge time (110, 120, and 130 seconds). The “*t*” test was then applied to the results obtained (results in Table 2). These results point to a method with acceptable accuracy and precision. Thus, the voltammetric method could be used for quantitative zalcitabine determination in medicaments. 

### 3.5. Determination of Zalcitabine in Pharmaceutical Formulations

The optimized voltammetric method was then used for the determination of zalcitabine in medicament. Twenty aliquots from this sample were, in parallel, analyzed by voltammetry. These results are shown in Table 3. In every analysis only the reduction zalcitabine peak potential appears at −1.18 V (other conditions as in Figure 4).

## 4. Conclusion

The differential pulse voltammetry proved to be a satisfactory method for the determination of zalcitabine in an acid medium (pH 2.0). Accordingly, the reduction zalcitabine peak potential appears at −1.18 V using drops of mercury as the working electrode. The application of this technique for the analysis of pharmaceutical formulations containing this substance produced statistically valid results, indicating that the voltammetric method may be an alternative in these analytical procedures. Various parameters studied, such as selectivity, linearity, precision, LOD, LOQ, accuracy, and robustness, showed also satisfactory results. In our laboratory we have been investigating the utilization of preadsorptive voltammetric techniques to the improvement of sensitivity and selectivity in the zalcitabine metabolites determination. The same voltammetric procedure also can be extended to many other medicaments used in the treatment of human immunodeficiency virus (HIV) infection: (i) the nucleoside reverse transcriptase inhibitors (NRTIs) zidovudine, didanosine, stavudine, lamivudine, abacavir, and emtricitabine; (ii) the nucleotide reverse transcriptase inhibitor (NtRTI) tenofovir disoproxil fumarate; (iii) the nonnucleoside reverse transcriptase inhibitors (NNRTIs) nevirapine, delavirdine, and efavirenz; (iv) the protease inhibitors saquinavir, ritonavir, indinavir, nelfinavir, amprenavir, lopinavir, and atazanavir [15–18].

## Figures and Tables

**Table 1:** Effect of pH on the differential pulse peak currents and potentials for 10 mg · L^−1^ of the zalcitabine in a solution of Briton-Robinson or Clark-Lubs buffer. Scan rate: 20 mV/s. Amplitude: 50 mV.

pH	*I*_*p*_ (nA)	−E_*p*_ (mV)
2.0	291	1190
2.5	307	1210
3.0	299	1230
3.5	260	1250
4.0	236	1270
4.5	222	1300
5.0	54	1360
5.5	70	1390

**Table 2:** Results obtained from validation of the voltammetric method.

Validation parameters	Results
Selectivity	Satisfactory (in accordance with the *F* and Student's tests)
Linearity	10.0–28.0 mg/L
Detection limit	2.08 mg/L
Quantification limit	4.12 mg/L
Repeatability	RSD = 0.82%
Intraday precision (different days)	Satisfactory (in accordance with the Cochran, *F*, and Student's tests)
Accuracy (recovery)	100.95%
Robustness	Only to purge time variations (110 or 130 seconds)

**Table 3:** Results obtained in the medicament.

Medicament	Measured quantities (mg/cap.)	Declared quantities (mg/cap.)	Standard deviation	Relative standard deviation (%)	Quantities obtained (%)
Zalcitabine	0.758	0.750	0.006216	0.82	101.07
